# Guidelines for the vaccination of HIV-infected adolescents and adults in South Africa

**DOI:** 10.4102/sajhivmed.v19i1.839

**Published:** 2018-05-23

**Authors:** Sipho K. Dlamini, Shabir A. Madhi, Rudzani Muloiwa, Anne von Gottberg, Mahomed-Yunus S. Moosa, Susan T. Meiring, Charles S. Wiysonge, Eric Hefer, Muhangwi B. Mulaudzi, James Nuttall, Michelle Moorhouse, Benjamin M. Kagina

**Affiliations:** 1 Department of Medicine, University of Cape Town, South Africa; 2 South African Medical Research Council, Respiratory and Meningeal Pathogens Research Unit, Faculty of Health Sciences, University of the Witwatersrand, South Africa; 3 Department of Science and National Research Foundation: Research Chair: Vaccine Preventable Diseases, Faculty of Health Sciences, University of the Witwatersrand, South Africa; 4 Department of Paediatrics and Child Health, University of Cape Town, South Africa; 5 Centre for Respiratory Diseases and Meningitis, National Institute for Communicable Diseases, a division of the National Health Laboratory Services, Johannesburg, South Africa; 6 School of Pathology, Faculty of Health Sciences, University of the Witwatersrand, Johannesburg, South Africa; 7 Department of Infectious Diseases, Division of Internal Medicine, Nelson R. Mandela School of Medicine, University of KwaZulu-Natal, South Africa; 8 National Institute for Communicable Diseases, Division of the National Laboratory Services, South Africa; 9 School of Public Health, University of the Witwatersrand, South Africa; 10 Cochrane South Africa, South African Medical Research Council, Division of Epidemiology and Biostatistics, Department of Global Health, Stellenbosch University, South Africa; 11 Division of Epidemiology and Biostatistics, School of Public Health and Family Medicine, University of Cape Town, South Africa; 12 Private Practice, Johannesburg, South Africa; 13 Private Practice, Rustenburg Phomolong Medical Centre, South Africa; 14 Wits Reproductive Health and HIV Institute, Johannesburg, South Africa; 15 School of Public Health and Family Medicine, University of Cape Town, South Africa; 16 Vaccines for Africa Initiative, University of Cape Town, South Africa

## Introduction

The introduction of vaccines has been one of the most cost-effective strategies to prevent infectious diseases in humans.^1^ It is estimated that vaccines save 2–3 million lives globally each year (WHO and UNICEF 2005). Vaccines are critical in preventing vaccine-preventable diseases (VPDs), particularly among immunocompromised individuals such as those with HIV infection. HIV-infected individuals have impaired host defence (cellular and humoral immunity), and hence have a greater risk and severity of VPDs than non-immunocompromised populations.^2^

Effective antiretroviral therapy (ART) has resulted in HIV infection becoming a chronic manageable illness. Despite ART, systemic inflammation and immune activation persist in HIV-infected individuals and are associated with adverse health outcomes.^3^ Furthermore, immune reconstitution is incomplete following ART initiation, which might include absence of underlying memory responses previously induced through natural infection or early childhood vaccination. Vaccination, and possibly revaccination, could be an important complement to ART in preventing further complications in this population.^4^

The impact of VPDs in HIV-infected individuals not only relates to the acute phase mortality but also derives from the high prevalence of these diseases in the HIV population, with effects on long-term morbidity and mortality.^5^

Vaccines enhance immunity against infections that may have been compromised following HIV infection. The benefits of vaccination in HIV-infected individuals must be weighed against its risks. However, in the absence of severe immunosuppression, the majority of routine vaccines, including live attenuated vaccines, have been found to be safe for use in HIV-infected individuals.^2^ At present, the evidence suggests that vaccination of HIV-infected persons with suppressed viral loads and nadir CD4+ counts greater than 200 cells/µL has little or no deleterious effect on immune and inflammatory activation.^4,6^ The duration of seroprotection for most vaccines has been shown to be shorter in HIV-infected patients compared to uninfected individuals.^7^ There are insufficient data to guide the optimal timing for revaccination or booster vaccination among HIV-infected individuals.^8^

There are no national guidelines for the use of vaccines for HIV-infected people in South Africa. Establishment of guidelines could contribute to improving vaccine uptake in this population. The national guidelines will also help healthcare workers in decision-making towards vaccinating HIV-infected individuals. The use of vaccines in the HIV-infected is generally safe and causes no harm among patients with CD4+ counts above 200 cells/µL and with very low viral load (< 50 copies/mL).^9,10^ Even among HIV-infected individuals who are severely immunocompromised, the majority of non-live vaccines are potentially safe, although the immunogenicity and effectiveness need to be elucidated.

As part of a comprehensive approach to the management of HIV-infected individuals, the Southern African HIV Clinicians Society aims to promote the use of vaccines in this group. Currently, there are no guidelines on the use of vaccines among the HIV-infected in South Africa. Furthermore, suboptimal knowledge among HIV-infected individuals and healthcare providers undermines the use of vaccines in a population that could otherwise benefit most from vaccination.

## Challenges of vaccination

Challenges faced by the healthcare providers wanting to vaccinate HIV-infected individuals include, firstly, a lack of national guidelines and policies. Secondly, vaccines are not generally available at settings where HIV-infected adults seek care. Furthermore, cost of the vaccines may be a limiting factor in the health system which is already burdened by various other costs. These barriers impact negatively on the uptake of vaccines among HIV-infected adolescents and adults.

## Rationale for the development of national guidelines

The epidemiology of HIV disease has changed significantly in the last 10 years in South Africa. More people with HIV are living longer as access to ART increases. Associated with this increase in life expectancy is an increased risk of VPDs despite suppressive ART.

## Scope of guideline

These national guidelines for vaccines available for HIV-infected adolescents and adults in South Africa have been developed to address challenges of adolescent and adult immunisation in the setting of HIV. The guidelines specifically focus on the most common VPDs in sub-Saharan Africa among HIV-infected adolescents and adults. The best available evidence has been used to make recommendations on the appropriate use of active and passive immunisation in HIV-infected adolescents and adults.

## Influenza vaccines

Influenza is a seasonal viral disease that often leads to a high morbidity and mortality both nationally and globally. The mortality from influenza in South Africa is between 6000 and 11 000 deaths every year.^11^ Individuals who are HIV-infected have a four- to eight-fold risk of influenza and are 1.5 times more likely to die as a result of this than HIV-uninfected individuals.^12,13^ There are several types of licensed vaccines against seasonal influenza; only the trivalent inactivated influenza vaccine (TIV) is currently available in South Africa. Vaccination against influenza has been shown to be an effective preventive strategy, and many international immunisation guidelines recommend vaccination against the seasonal influenza in HIV-infected individuals. Evidence suggests that HIV-infected individuals may have reduced immune responses to influenza vaccines, especially when CD4+ cell counts are below 200 cells/µL.^14^ Despite this, influenza vaccination is effective in preventing influenza infection and reducing severity of influenza-associated illnesses among HIV-infected persons.^10^ Inactivated influenza vaccines administered to HIV-infected individuals have been found to be safe with a vaccine efficacy of approximately 75% in South Africa, despite a relatively modest immune response as measured by the haemagglutinin inhibition (HAI) assay.^15^ Similarly, despite low immunogenicity per HAI assay of influenza vaccine in HIV-infected compared to HIV-uninfected pregnant women in South Africa, the vaccine efficacy was 73% against influenza confirmed illness among HIV-infected women.^16^ These studies suggest that the conventional immune read-outs which we use for predicting vaccine efficacy for inactivated influenza vaccine, that is, HAI titres ≥ 40, might not be useful and may underestimate vaccine efficacy in this population.

The influenza season in South Africa is variable. It can start as early as April or as late as July and lasts between 12 and 25 weeks.^17^

The panel recommends that the trivalent- or quadrivalent-inactivated influenza vaccine be administered between March and May each year to all HIV-infected persons, irrespective of CD4+ cell count, HIV viral load or pregnancy status.

## Pneumococcal vaccines

Invasive pneumococcal disease (IPD) in HIV-infected individuals is a significant cause of morbidity and mortality.^18^ Even with improved and increased access to universal ART in South Africa, the relative risk of having IPD among the HIV-infected is 35–100 times more than HIV-uninfected individuals.^19,20,21^ Available evidence from Africa suggests that pneumococcal conjugate vaccines (PCV) are effective against the vaccine-type pneumococcal disease when given to HIV-infected adults.^19,20^ In South Africa, PCV has been included in the routine childhood immunisation programme since 2009. Early post-vaccine impact data from South Africa showed a 40% reduction in vaccine-type IPD over non-vaccine-type IPD in HIV-infected adults, indicating that there is some indirect protection to unvaccinated adults.^22^

However, with such a high burden of disease in the HIV-infected population, there is a need to consider additional or complementary strategies to prevent pneumococcal disease in HIV-infected adults. An example would be a combination vaccine strategy of the 13-valent pneumococcal conjugate vaccine (PCV13) and 23-valent pneumococcal polysaccharide vaccine (PPV23). This directly increases protection to serotypes that are in PPV23 but not in PCV13.

Broadly, there are two types of vaccine available for use to prevent pneumococcal disease: the pneumococcal polysaccharide vaccines (PPV) and the PCV. The PPV23 is protective against all-cause pneumonia and pneumococcal disease in HIV-infected adults, although data vary as to whether the protective benefit is irrespective of CD4+ count above a particular threshold, or dependent on HIV viral load at time of vaccination.^23^ Results from a randomised placebo-controlled trial of PPV in HIV-infected subjects conducted in Uganda found an increase in all-cause pneumonia in the vaccine arm.^24^ As a result, some international guidelines do not recommend vaccination with PPV among HIV-infected adults with CD4+ counts less than 200 cells/µL. The immunological and clinical efficacy of PPV23 for HIV-infected adults, particularly in the absence of ART, remains controversial.^19^

A randomised double-blind placebo-controlled trial of PCV7 in HIV-infected adults in Malawi showed that the vaccine was protective against recurrent IPD.^19^ Other studies have confirmed the safety and immunogenicity of PCV13 in HIV-infected adults.^20^ The use of PCV in HIV infection is supported by studies showing prevention of pneumococcal pneumonia in children and prevention of recurrent IPD in adults.^25^

The use of either PCVs or PPVs will be a balance between the resources available to pay for the vaccine and the evidence to support the use of either of the vaccines or even a combination. The evidence suggests that the burden of pneumococcal disease is high in HIV-infected individuals, and vaccination against pneumococcal disease is an important strategy to reduce this.^26^ Globally, various advisory committees on vaccination differ in their recommendations for vaccination against pneumococcus.^27^ As an example, the United States Advisory Committee on Immunisation Practices (ACIP) and Infectious Diseases Society of America (IDSA) guidelines recommend PCV13 followed by PPV23 in HIV-infected adults; however, the evidence supporting this is limited.^25^ In contrast, the British guideline recommends vaccination with PCV13 only, irrespective of CD4+ count, viral load or ART use.^28^ Available evidence suggests that the use of PPV23 or PCV13 is safe in HIV-infected individuals on ART or with a CD4+ count greater than 200 cells/µL.^8,29^

Vaccination against pneumococcal disease should be recommended for all HIV-infected individuals regardless of CD4+ count, but ideally when HIV viral load is < 1000 copies/mL.^29^ This guideline supports the prime-boost immunisation approach of first vaccinating with PCV13 followed by PPV23 eight weeks later. Alternatively, PCV13 can be used alone if available. For those who are to receive PPV23 as the primary vaccine, it is suggested that the PPV23 vaccine be given to those who have achieved ART-driven virologic suppression, regardless of the CD4+ count.

## Meningococcal vaccines

*Neisseria meningitidis* is endemic in South Africa, presenting as meningococcal bacteraemia or meningitis. HIV infection is an important risk factor for acquiring invasive meningococcal disease (IMD), with a relative risk 5–13 times greater than the general population.^30,31,32,33^ Epidemiological data from South Africa show an increased case-fatality rate of IMD in HIV-infected patients (20% vs. 11% in HIV-uninfected patients) that could be explained by their increased odds of bacteraemia compared to meningitis.^33^

Currently in South Africa, meningococcal disease is a minor contributor to mortality among the HIV-infected, when compared to other infections such as tuberculosis (TB) and pneumococcal disease.^34^ However, the availability of effective vaccines against meningococcal disease should warrant the use of these vaccines in HIV-infected adults where needed. In 2016, the ACIP added HIV infection as a high-risk condition for meningococcal disease, based on the growing body of evidence supporting an increased risk of meningococcal disease in HIV-infected individuals.^30,35^ Prospective cohort studies or case-controlled studies are needed to evaluate this association and further clarify the level of immunosuppression at which the increased risk occurs.

Our recommendation is that, where possible, vaccination should be considered in HIV-infected adults with a CD4+ count above 200 cells/µL. Where resources are limited, routine meningococcal vaccination of HIV-infected adults should follow the indications for meningococcal vaccination for the general population.

The general indications for vaccine are:

- functional or anatomic asplenia
- complement deficiency
- individuals at risk of exposure through travel or work settings (routine microbiology laboratories)
- individuals living in hostels, or university or college residences
- individuals at risk through an outbreak, including men who have sex with men (MSM) who may be exposed to outbreak strains because of social interactions within the global MSM community.

Two quadrivalent meningococcal vaccines covering serogroups A, C, W135 and Y are currently available in South Africa. One is a polysaccharide vaccine and the other is a protein-conjugated polysaccharide vaccine. Because of the hypo-immunity associated with repeated doses of the plain polysaccharide vaccine, it is recommended that HIV-infected individuals receive the conjugate vaccine.^36^ A two-dose primary schedule (given 8–12 weeks apart) is recommended to increase the likelihood of a protective primary immune response. This should be followed by booster doses every five years. HIV-infected persons should ideally be vaccinated before their CD4+ cell percent drops to < 25%.^37^

## Pertussis vaccines

There are currently two categories of pertussis vaccines, namely, acellular and whole-cell vaccines.^38^ Acellular vaccines are developed from one or more highly purified individual pertussis antigens, and whole-cell vaccines are based on killed *Bordetella pertussis* organisms. Although infants below three months of age are at the highest risk of death from *Bordetella pertussis*, current epidemiological evidence shows that it is an important cause of respiratory disease in both adolescents and adults.^38,39,40^ Despite the availability of vaccines and adequate coverage of childhood vaccinations, incidence of the disease continues to increase in adolescents and adults, possibly because of waning immunity. There are very few studies of adolescent and adult pertussis in the HIV-infected population, with most of the data on pertussis coming from children.^41^ Maternal carriage, HIV exposure of young infants, and incomplete vaccination seem to be the most important risk factors for childhood pertussis. Available evidence indicates that maternal vaccination offers significant protective benefits to both mothers and infants.^39^

Evidence shows that asymptomatic pertussis infection is more common than symptomatic infection in healthy adolescents and adults.^42^ More data are needed to understand the epidemiology and the burden of pertussis in countries where HIV/AIDS is endemic. To date, there are no studies with regard to the immunogenicity or efficacy of pertussis vaccination in HIV-infected adults. Evidence from studies in children demonstrates that HIV infection lowers antibody response following vaccination, and high CD4+ counts at vaccination improve antibody response.^41^ With the lack of data on the burden of disease in HIV-infected adolescents and adults, the recommendation would be that only pregnant women, regardless of CD4+ counts or viral load, be vaccinated (with acellular vaccine) during each pregnancy until there is more evidence to do otherwise. This is mainly to increase their antibody levels, so as to enhance transplacental antibody transfer to their newborns, who are at high risk of pertussis illness.^43^

## Diphtheria and tetanus vaccines

Recommendations for the use of tetanus and diphtheria vaccines in HIV-infected adults mirror that for the general population. Both vaccines are inactivated toxoid and are therefore safe for use in HIV-infected individuals. Vaccine-induced immune responses following tetanus vaccination appear to be similar in both HIV-infected and HIV-uninfected individuals.^44^ In contrast, vaccine-induced immune responses following diphtheria vaccination are lower in the presence of HIV infection and are influenced by the CD4+ counts.^7^ Studies show that following primary vaccination with tetanus and diphtheria vaccines among HIV-infected children, there is a quick waning of the vaccine-induced immunity.^45^

However, as the evidence is lacking on the optimal booster schedule for HIV-infected adults, the recommendation is administration of tetanus and diphtheria vaccines at least every 10 years until more data are available.

## Hepatitis A vaccines

Hepatitis A virus (HAV) is the most common cause of acute viral hepatitis, with approximately 1.4 million clinical cases annually.^46^ The disease burden is most likely underestimated because of a high incidence asymptomatic infection. The main route of infection is faecal-oral, and infection is driven by poor access to safe water and sanitation services. In Africa, data on HAV infection are limited but the continent is considered to be a highly endemic setting.^47^ In such settings, routine vaccination in childhood is not recommended because of immunity acquired following natural infection.^47^ Data from South Africa show that antibodies to HAV in Black African adults are almost 100% by the age of 20 years, whereas only 30% – 40% of White adults are positive for HAV antibodies by the age of 20 years.^47^

Men who have sex with men are at a higher risk of acquiring HAV infection with reported outbreaks in some parts of the world.^7,48^ The burden of disease in this population group is 1.5–3 times that of the general population especially in the developed world.^49^

About 13 studies have reported data on HAV vaccination of HIV-infected adults.^46^ All these studies explored the immune responses to HAV vaccination and reported that the vaccine is safe and without effect on the clinical progression of HIV infection among adults on ART.^46^ Vaccination at higher CD4+ counts is associated with better vaccine-induced immune responses. However, given the heterogeneity of the methodology and study populations, these results should be interpreted with caution.^46^

There is no consensus on the benefits of routine vaccination of HIV-infected adults with HAV vaccines. Advisory groups such as ACIP and the WHO Strategic Advisory Group of Experts on Immunisation (SAGE) recommend vaccination in the presence of other risk factors (medical, behavioural, epidemiological or occupational risk factors). High risk groups for HAV infection typically include MSM, injection drug users, people travelling to or working in countries with a high or intermediate endemicity of HAV, people with chronic liver disease (including hepatitis B or C), bleeding diathesis, immunosuppressed individuals who have undergone transplantation and contacts of children arriving from countries with high or intermediate HAV endemicity.^46^

We recommend that HAV vaccination should be given if there are other medical, behavioural, epidemiological or occupational conditions in addition to HIV infection. The accepted schedule is two doses separated by 6–12 months.^46^

## Hepatitis B vaccines

Hepatitis B virus (HBV) vaccination is highly recommended for individuals with HIV infection. Globally, the introduction of routine HBV vaccination has resulted in a significant decrease of HBV infection and the overall disease burden.^46,50^ Co-infection with HBV and HIV is considered endemic in sub-Saharan Africa, including South Africa. Both viruses share the same routes of infection, and it is estimated that the prevalence of chronic HBV in HIV-infected individuals ranges from 0.4% to 23% in South Africa.^51^ The overall global prevalence of co-infection is around 10%, but this estimate varies from region to region. The rates of HBV infection among HIV-infected individuals are estimated to be 20 times higher than among HIV-uninfected persons.^46,52^

In South Africa, HBV vaccination has been part of the childhood immunisation programme since April 1995. Although vaccination coverage against HBV in childhood is high,^53^ many adults born before 1995 were not vaccinated earlier in life and remain at high risk of HBV infection.^54^

The administration of vaccines against HBV among HIV-infected individuals has been shown to be safe in many studies.^55^ However, immunologic responses are diminished.^46^ A successful response to HBV vaccination is most frequently associated with an undetectable HIV viral load and a CD4+ count above 200 cells/µL.^56^

The international recommendation for administration of vaccines against HBV is a four-double-dose regimen of a recombinant HBV vaccine (40 µg) at 0, 1, 2 and 6 months.^57^ However, the WHO recommendation is a three-dose regimen of recombinant HBV vaccine (20 µg) at 0, 1 and 6 months.^55^ The double-dose regimen induces higher peak anti-HBs antibody titres than the standard-dose regimen, but no clear difference in the proportion of adults with protective antibodies up to five years after vaccination.^8,58,59,60^ The British HIV Association (BHIVA) and the ACIP both recommend serologic antibody testing one month and six to eight weeks, respectively, following administration of the last dose of the vaccine.^46^

The panel supports the four-double-dose regimen HBV vaccine schedule. If this is not possible, then the standard regimen should be administered. The panel does not recommend serologic antibody testing and is only advised for healthcare workers or others at high risk.

## Human papilloma virus vaccines

Human papilloma virus (HPV) infection is a common infection in HIV-infected adults. The virus is more prevalent and persistent in HIV-infected adults. The persistence of certain serotypes of HPV infection is associated with squamous dysplasia and cancer.^61^ In 2008, it was estimated that of the 12.7 million new cancers that occurred worldwide, around 5% were attributable to HPV infection.^61,62^ The highest burden of HPV infection and associated diseases is carried by women.^61,63^

HPV vaccines are safe and immunogenic in HIV-infected adults.^64^ As observed with other vaccines, HPV vaccine-induced immune responses are better in HIV-infected subjects with CD4+ cell counts greater than 200 cells/µL and suppressed viral loads. There are limited data on the efficacy and durability of protection provided by HPV vaccines in HIV-infected adults, whether vaccinated before or after acquisition of HIV infection. There is also no evidence to guide booster vaccine dosing in HIV-infected individuals.^61^

In South Africa, HPV vaccination is routinely recommended for preteen girls aged 9–13 years, regardless of HIV status. HPV vaccination is recommended for all HIV-infected adult men and women, and MSM aged up to 40 years, regardless of CD4+ count, ART use or viral load. In the public sector in South Africa, the bivalent vaccine is currently in use. In the private sector, the quadrivalent HPV vaccine (4vHPV) is also available. The 4vHPV was originally tested and approved as a three-dose regimen, with a dosing schedule of 0, 2 and 6 months. More recently, a two-dose schedule (6 or 12 months apart) has been recommended by the World Health Organization for younger age groups (e.g. 9–14 years at first dose). This is because immunogenicity with two doses in preadolescent and early adolescent girls was noninferior to antibody responses in women aged 16–26 years receiving three doses.^65,66^ Should the vaccine schedule be interrupted, it is recommended that the vaccination series be completed, rather than restarted.^28^

## Poliovirus vaccines

There are very little data on wild-type poliomyelitis in HIV-infected individuals. Poliomyelitis is exceedingly rare in South Africa but continues to occur in a few countries in the world. The live attenuated oral polio vaccine should be avoided in immunocompromised HIV-infected individuals because of the potential risk of paralytic polio.^67^ Hence, the inactivated vaccine is recommended.

As with many other vaccines, HIV-infected adults have reduced vaccine-induced immune response to inactivated polio vaccine,^44,68^ which is better if the CD4+ cell count is above 200 cells/µL.

We recommend that all unvaccinated HIV-infected adults be vaccinated with the inactivated polio vaccine, regardless of CD4+ count, especially if the individuals are travelling to high-risk regions (such as Nigeria, Pakistan and Afghanistan). Three doses should be administered at 0, 1–2 and 6–12 months with a further dose after 10 years if at high risk.^69^

## Live vaccines

The use of live vaccines is contraindicated in HIV-infected adults, especially if the CD4+ count is below 200 cells/µL, and/or there is clinical evidence of acquired immune deficiency syndrome (AIDS). There is evidence that for some live vaccines, there is a potential for the proliferation of vaccine-related viruses in the host, varicella as an example.^70,71^

## Measles, mumps and rubella vaccine

Measles, mumps and rubella (MMR) vaccines are live and therefore contraindicated in individuals with CD4+ counts below 200 cells/µL.

The ACIP has since 2013 made recommendation about the use of MMR vaccine in HIV-infected adults, provided the CD4+ counts are above 200 cells/µL, without laboratory evidence of immunity or past disease.^72^ A retrospective study from the USA assessing the MMR serostatus of HIV-infected adults reported that 15% of HIV-infected adults are seronegative. A seronegative MMR status was associated with a longer duration of HIV infection, younger age and those born after 1957. This study also reported that about 50% of HIV-infected adults seroconverted after MMR vaccination. This study found that MMR vaccination did not lead to progression of HIV disease, nor did it impact on HIV disease control.^72^ This study therefore demonstrated the safety and efficacy of MMR vaccination in HIV-infected adults with CD4+ counts above 200 cells/µL and undetectable viral loads.

Most international guidelines recommend MMR vaccination in non-immune adults infected with HIV with a CD4+ cell count above 200 cells/µL.^28,72^

## Varicella vaccines

It is estimated that 4.2 million cases of severe varicella infection globally result in hospitalisation or death. Varicella zoster virus (VZV) infections and related deaths are common in HIV-infected individuals. Most HIV-infected adults will have evidence of prior infection or immunity. There is an effective vaccine for varicella which is in use in high-income countries. There are limited data on varicella vaccination in HIV-infected adolescents or adults, although one study has reported on the safety and modest immunogenicity of two doses of varicella vaccine in HIV-infected adults with a CD4+ count above 400 cells/µL with a suppressed viral load.^73^ At present in Africa, there is a lack of varicella epidemiology and socio-economic data on the impact of this disease. Therefore, there are no data on which to base recommendations regarding the use of this vaccine in HIV-infected adults.^74^

## Zoster vaccine

Reactivation of latent varicella-zoster virus infection leads to a debilitating condition: *Herpes zoster* (HZ; known as shingles). Despite the increased uptake and use of ART, the incidence of *Herpes zoster* remains increased in HIV-infected adults. The increased uptake of ART has also resulted in a greater proportion of HIV-infected people living beyond the age of 50 years.^7,75,76^

The use of varicella vaccine is recommended in varicella-susceptible adults, as long as they have a CD4+ count above 200 cells/µL; the same CD4+ threshold is used for MMR and yellow fever vaccines. No transmission of vaccine strain VZV has been documented in people with HIV infection with a CD4+ count above this threshold. The administration of live attenuated *Herpes zoster* vaccine (LAHZV) to 295 HIV-infected adults with a CD4+ count above 200 cells/µL was found to be safe and immunogenic, with no cases of vaccine strain infection.^75,77^

At present, there are no epidemiological data to support the use of this vaccine in Africa. There is a systematic review underway to assess the morbidity and mortality of VZV infection.^74^ Until more evidence becomes available, we do not recommend this vaccine for HIV-infected adults.

## Conclusion

The role out and increased uptake of ART has led to improvements in mortality of HIV-infected individuals. A great proportion of individuals are still at risk of vaccine preventable infections. Vaccines are an important tool in protecting individuals at risk of vaccine preventable infections. A suppressed viral load and a CD4+ count above 200 cells/µL improve immunogenicity to vaccines.

These guidelines are primarily aimed to help clinicians who look after people living with HIV infection. For some vaccines, there may not be enough local evidence to give guidance. It is believed that by producing this guideline, the gaps in our local data will generate research to provide the necessary evidence. This guideline attempts to take into account our local epidemiological situation in formulating our guidance where possible (see Table 1).

**TABLE 1:** Vaccination guidelines for HIV-infected adolescents and adults.

Vaccine	Indication	Safety CD4+ count	Doses for unvaccinated adults	Booster	Comments
MMR vaccine	Measles, mumps or rubella seronegative	➢ 200 cells/mL	2 doses (28 days apart)	Protection likely lifelong	Mainly indicated in measles seronegative HIV-infected women of childbearing age
					Pregnancy should be avoided for 1 month after vaccination
Influenza	R	Any	1 dose	Yearly	-
Pneumococcal	R	Any	1 dose	-	Given with PPV23 but must be given first
Conjugated (PCV13)					
Pneumococcal	RS	➢ 200 cells/mL	1 dose	5–10 years	Given with PCV13 but given 8 weeks after PCV13
Polysaccharide (PPV23)					Can be given to patients with CD4 count < 200 cells/mL if on ART and VL suppressed
					Maximum 2 booster doses, 1 booster dose in patients > 65 years. Poor response if CD4+ cell count < 200 cells/mL and VL not suppressed
Hepatitis B	R	Any	4 doses (40 µg) or 3 doses (20 µg)	Not clear awaiting evidence	-
Hepatitis A	RS – travel, MSM, liver disease	➢ 200 cells/mL	2 doses	10 years	-
Meningococcal	RS	Any	2 doses	5 years	-
Tetanus-diphtheria (Td)	R	Any	-	10 years	-
Pertussis-acellular	R	Any	1 dose	10 years	Given in pregnancy combined with tetanus-diphtheria (DTPa/dTpa)
Poliomyelitis-inactivated	RS	➢ 200 cells/mL	3 doses	none	-
Human papilloma virus (HPV)	RS – females, MSM	Any	2 doses	none	-
Varicella	NR	-	-	-	May be considered if CD4+ count > 400 cells/mL
Zoster	RS	≥ 200 cells/mL	1 dose	none	Only use if CD4+ count ≥ 200 cells/µL

MMR, measles, mumps, and rubella; R, recommended; RS, recommended in selected individuals; NR, not recommended; VL, viral load; HBsAb, hepatitis B surface antibody; MSM, men who have sex with men.
